# DDT and Malaria Prevention

**DOI:** 10.1289/ehp.0901276

**Published:** 2010-01

**Authors:** Richard Tren, Donald Roberts

**Affiliations:** Africa Fighting Malaria, Washington, DC, E-mail: rtren@fightingmalaria.org; Uniformed Services University of the Health Sciences, Bethesda, Maryland

In his commentary “Global Status of DDT and Its Alternatives for Use in Vector Control to Prevent Disease,” van den Berg (2009) raised concerns about the impact of DDT (dichlorodiphenyltrichloroethane) and its derivates on human health, in spite of the fact that DDT has been used widely for seven decades and no properly replicated and confirmed study has found any specific human health harm. Given the enormous and proven public health benefits arising from the use of DDT in disease control, it is incumbent on van den Berg to cite human health harm from DDT exposure that fulfills the basic epidemiologic criteria for a clear and unambiguous cause-and-effect relationship. In the absence of such evidence, van den Berg’s concerns should be ignored.

van den Berg reported on high levels of human exposure to DDT among those living in sprayed houses but presented no evidence of actual human harm arising from that exposure. van den Berg is not the first to consider this issue. Many prestigious and authoritative groups and individuals with no ideologic agenda have evaluated potential DDT harms over the last 70 years, and have consistently found no evidence of DDT harm that would cancel out the enormous health benefits of its use in malaria-endemic countries. Yet van den Berg (2009) stated that “initial work suggests that nonoccupational exposure through (IRS) is associated with impaired semen quality in men” but failed to mention that the association is exceedingly weak. In addition, van den Berg neglected to report on the evidence of growing populations wherever DDT has been used in malaria control, a fact that would undermine the idea that DDT significantly harms human fertility.

With respect to other health effects, such as early pregnancy loss, fertility loss, leukemia, and various cancers, van den Berg (2009) admitted that in “many cases the results have not been consistent between studies,” but he went on to state that “these accumulating reports bear much concern, particularly in relation to chronic effects.” This is a stunning embrace of belief over science. van den Berg’s conclusion defeats the very purpose of epidemiologic standards for decisions about cause–effect relationships. Accumulations of data and results of distinct studies that provide weak or no evidence of harm to human health do not argue for a hidden cause–effect relationship; they argue instead that no cause–effect relationship exists.

Where DDT has been used in malaria control over many decades, populations have grown and health outcomes have improved. Yet van den Berg (2009) stated, without reference, that “a gain in infant survival resulting from malaria control could be partly offset by an increase in preterm birth and decreased lactation, both of which are high risk factors for infant mortality in developing countries.”

van den Berg addressed the potential environmental harm that could be caused by DDT use in malaria control. Although he noted that DDT is sprayed indoors for malaria control and is used in small quantities, van den Berg (2009) stated that “DDT sprayed indoors may end up in the environment.” If some residues are found outdoors, they will be demonstrably concentrated in soil within the first few meters from the house. Documentation from many studies shows that DDT movement is likely to be so small and so gradual as to be insignificant (Smith and Webley 1969; Viera et al. 2001). DDT use in malaria control is by definition used indoors, whereas environmental management, which is promoted as an alternative to the use of insecticides for malaria control, is by definition performed outdoors (e.g., larvivorous fish, drainage of wetlands). If van den Berg has evidence to show that small uses of DDT sprayed indoors is more harmful than massive environmental changes outdoors, he should have included it in his review.

van den Berg’s discussion of insecticide resistance and repellency and irritancy of DDT is both confused and confusing. Citing a personal communication, van den Berg (2009) wrote that “the development and spread of insecticide resistance is much slower when vector populations are under effective control . . . suggesting that suppressing vector proliferation helps prevent or delay the development of resistance.” To conclude that insecticide resistance is forestalled by using insecticides to suppress vectors is absurd. It is a demonstrable fact that spraying insecticides to kill insects will select for resistance. van den Berg seemed to suggest that DDT’s repellent actions may contribute to vector resistance, but he is wrong. Repellent action does not lead to toxic resistance because repellency reduces mortality, and it is mortality that exerts selective pressure for resistance.

Alternatives to DDT are always welcome. van den Berg (2009) correctly noted that operational capacity is a barrier to introducing these alternatives and that many countries have implemented health sector reforms that have decentralized decision making, planning, and budgeting. He relied on case studies (Barat 2006) of four countries to argue that decentralization can benefit malaria control. However, elsewhere, these case studies have been criticized and shown to be based on unreliable and even false data (Attaran et al. 2006). IRS and other malaria control operations require, to a significant degree, centralized decision making, planning, and budgeting. The trend of decentralization since the 1970s has not only limited the scope of IRS but has also been a factor contributing to the gradual increase in malaria transmission around the world. In addition, as documented by the World Health Organization (1986), decentralization was vigorously opposed by many malaria scientists. It is unreasonable to argue that the very factors that undermined IRS programs are reasons to further limit IRS.

van den Berg (2009) concluded that “environmental management and other nonchemical methods within [integrated vector management] strategies . . . will increase the sustainability of control efforts and assist in achieving malaria elimination objectives.” There is little evidence to support such a statement; in fact, the supposed solutions proffered and their modes of delivery have contributed to the weakening of malaria control programs and the global increase in malaria. After almost 70 years of use, DDT—when used in IRS programs—remains one of the safest and most effective methods of saving lives from malaria. van den Berg’s assessment makes no constructive contribution to advancing the goal of controlling a very preventable disease.
